# Hendra Virus Vaccine, a One Health Approach to Protecting Horse, Human, and Environmental Health

**DOI:** 10.3201/eid2003.131159

**Published:** 2014-03

**Authors:** Deborah Middleton, Jackie Pallister, Reuben Klein, Yan-Ru Feng, Jessica Haining, Rachel Arkinstall, Leah Frazer, Jin-An Huang, Nigel Edwards, Mark Wareing, Martin Elhay, Zia Hashmi, John Bingham, Manabu Yamada, Dayna Johnson, John White, Adam Foord, Hans G. Heine, Glenn A. Marsh, Christopher C. Broder, Lin-Fa Wang

**Affiliations:** CSIRO Australian Animal Health Laboratory, Geelong, Victoria, Australia (D. Middleton, J. Pallister, R. Klein, J. Haining, R. Arkinstall, L. Frazer, J. Bingham, D. Johnson, J. White, A. Foord, H.G. Heine, G.A. Marsh, L.-F. Wang);; Uniformed Services University, Bethesda, Maryland, USA (Y.-R. Feng, C.C. Broder); Zoetis Research & Manufacturing Pty Ltd, Parkville, Victoria, Australia (J.-A. Huang, N. Edwards, M. Wareing, M. Elhay, Z. Hashmi);; National Institute of Animal Health, Ibaraki, Japan (M. Yamada);; Duke–NUS (Duke and the National University of Singapore) Graduate Medical School, Singapore (L.-F. Wang)

**Keywords:** Hendra virus, HeV, vaccine, HeV vaccine, One Health, G glycoprotein, zoonoses, viruses, zoonotic paramyxovirus, flying foxes, horses, humans, Pteropus bats, Australia, environment, vaccination

## Abstract

In recent years, the emergence of several highly pathogenic zoonotic diseases in humans has led to a renewed emphasis on the interconnectedness of human, animal, and environmental health, otherwise known as One Health. For example, Hendra virus (HeV), a zoonotic paramyxovirus, was discovered in 1994, and since then, infections have occurred in 7 humans, each of whom had a strong epidemiologic link to similarly affected horses. As a consequence of these outbreaks, eradication of bat populations was discussed, despite their crucial environmental roles in pollination and reduction of the insect population. We describe the development and evaluation of a vaccine for horses with the potential for breaking the chain of HeV transmission from bats to horses to humans, thereby protecting horse, human, and environmental health. The HeV vaccine for horses is a key example of a One Health approach to the control of human disease.

Hendra virus (HeV) is an emerging zoonotic paramyxovirus for which natural reservoirs are the 4 species of flying fox (*Pteropus* bats) found on mainland Australia (*1*). HeV was discovered in 1994, and since then, infections have occurred in 7 humans, 4 of whom died. Each case-patient had a strong epidemiologic connection to similarly affected horses through exposure to equine secretions late in the incubation period, during terminal illness, or at the time of postmortem examination of infected animals (*2*): no human case of HeV infection has been attributable to direct spillover from bats (*3*). 

HeV infection in the bat host appears to be asymptomatic (*1*); however, in humans and horses there is evidence of initial virus replication in the nasopharynx that progresses through a viremic phase during which the virus spreads to major organ systems, resulting in disseminated endothelial cell infection, vasculitis, encephalitis, and pneumonia (*4*–*7*). There is no licensed anti-HeV therapeutic drug for use in any species. Experimental exposure of horses to HeV/Australia/Horse/2008/Redlands under Biosafety Level 4 (BSL-4) conditions identified comparatively low gene copy numbers in nasal secretions early in the incubation period. However, gene copy numbers increased exponentially with the onset of fever, when viral genome could also be recovered from blood, oral secretions, urine, and feces (*6*). Rapid progression of clinical signs, as observed in equine field cases of this disease, led to euthanasia of experimental animals on humane grounds. Viral RNA was recovered from all tissues sampled at postmortem examination, and virus was reisolated from lung, brain, lymphoid tissues, and kidney (*6*). In accordance with epidemiologic observations (*2*), it was concluded that HeV-infected horses in the immediate presymptomatic or symptomatic stages of disease pose a high risk for transmission of HeV to humans. This risk is then exacerbated because it is symptomatic horses that come to the attention of veterinarians, leading to various clinical investigations (e.g., respiratory tract endoscopy) that may facilitate human exposure to virus.

During 1994–2010, there were a total of 14 HeV outbreaks, including those with the 7 human infections. Then, in 2011, for reasons that are as yet poorly understood, an unprecedented 18 equine incidents, some involving >1 horse, occurred within a 3-month period and over an expanded geographic range, emphasizing that HeV was an unmanaged emerging disease (*3*). These events were accompanied by a marked rise in the number of HeV-related media reports. The reports had an increasingly politicized focus on the role (and control) of flying foxes as carriers of HeV (*8*) and a deemphasis of the critical role played by horses in HeV transmission to humans.

Heightened public awareness of the risk that infected horses posed to humans persisted and was paralleled by increased numbers of veterinarians leaving equine practice because of personal safety and liability concerns (*9*). The considerable investment in education and improved infection control measures that had been implemented did not effectively mitigate perceptions around the risks associated with the routine veterinary care of horses (*10*).

The actual mechanism of HeV transmission from bats to horses is probably complex and dependent upon socioeconomic, environmental, and ecologic factors (*11*), and there is currently no straightforward solution for preventing transmission. Eradication of flying foxes would pose extraordinary operational challenges, notwithstanding attendant moral, ethical, and environmental issues, and eliminating the interface between bats and horses is impractical for periurban and rural communities.

The most direct approach for reducing the risk posed to humans by HeV-infected horses would be implementation of a strategy that will lead to suppression of virus replication in horses. We describe the development and evaluation of a vaccine for horses with the potential for breaking the chain of HeV transmission from bats to horses to humans, thereby protecting horse and human health. The emergence of several highly pathogenic zoonotic diseases in humans in recent years has led to a renewed emphasis on the interconnectedness of human, animal, and environmental health, otherwise known as One Health. The HeV vaccine for horses, Equivac HeV (Zoetis, Parkville, VIC., Australia), is a key example of a One Health approach to the control of human disease (*12*).

## Materials and Methods

### Animals, Accommodation, Handling, and Biosafety

For efficacy studies, up to 3 female horses at a time were housed in single pens under BSL-4 conditions meeting the Victorian Bureau of Animal Welfare Code of Practice for the Welfare of Horses (www.dpi.vic.gov.au/agriculture/about-agriculture/legislation-regulation/animal-welfare-legislation/codes-of-practice-animal-welfare/code-welfare-of-horses). One of the sides of each pen was able to be moved in toward the horse on a ratchet mechanism, allowing staff close access to the horses, as required, over the side of the pen without the need for them to enter the pen itself (*13*). Room temperature was maintained at 22°C with 15 air changes/h; humidity ranged from 40% to 60%. Horses were fed a mixture of lucerne (alfalfa) and grass hay, concentrates, and specified fruit and vegetables. On the day before HeV exposure, an indwelling jugular catheter was sutured in position, and an intrauterine temperature data-logger was placed into each horse. All vaccinated horses were euthanized electively on day 7, 8, or 9 after challenge; unvaccinated horses were euthanized upon reaching a predetermined humane endpoint (6–9 days after vaccination). The humane end point was defined as fever for up to 48 h accompanied by increased respiratory rate, dyspnea, depression, ataxia, or pressing the head against the side of the stall. Euthanasia was conducted by intravenous injection of a barbiturate following sedation with intravenous detomidine and butorphanol.

Ferrets and guinea pigs used as controls in efficacy studies to confirm pathogenicity of the inoculum were housed in pairs in the BSL-4 facility, given species-appropriate dry rations and dietary treats, and provided with water ad libitum. For virus challenge and sampling, they were immobilized by intramuscular injection of a mixture of ketamine hydrochloride (3 mg/kg) and medetomidine (30 g/kg). The effects of medetomidine were reversed by intramuscular injection of atipemazole (15 g/kg). While in the BSL-4 animal room, staff wore fully encapsulated suits with an external air supply.

As appropriate, animal studies were endorsed by the CSIRO Australian Animal Health Laboratory Animal Ethics Committee and/or Commonwealth Serum Laboratories /Zoetis Animal Ethics Committee. Work using gamma-irradiated HeV soluble G (HeVsG) glycoprotein produced in Chinese hamster ovary (CHO) cells was done under Australian Quarantine and Inspection Service in vivo permit number 2012/012, and work using non-gamma–irradiated HeVsG glycoprotein produced in 293F human embryonic kidney was done under Australian Quarantine and Inspection Service in vivo permit number 2010/027. All clinical trials were conducted under the Australian Pesticides and Veterinary Medicines Authority research permits PER 7250, PER 13169, PER 13247, and PER 13418.

### Vaccine Preparation

A subunit vaccine containing recombinant HeVsG glycoprotein (*14*) was formulated in a proprietary adjuvant (Zoetis). For vaccine formulation, HeVsG glycoprotein was produced by using a Chinese hamster ovary (CHO) or a 293F human embryonic kidney cell expression system (*15*) with 1 of 2 different HeVsG glycoprotein preparations: 1) affinity-purified sG glycoprotein (293F cells) or 2) clarified sG containing cell culture supernatant (CHO cells). Vaccines for initial efficacy studies in target species were formulated with 50 μg or 100 μg of affinity-purified sG glycoprotein. All subsequent vaccines were formulated with clarified CHO cell culture supernatant that was then gamma irradiated. The change of the expression system from 293F cells to CHO cells was driven by the need for higher antigen yields, and equivalence was supported by laboratory analysis of the expressed antigens from the 2 systems and a comparison study in ferrets. Vaccine formulations used in efficacy studies are summarized in Table 1.

**Table 1:** Details, by efficacy trial number, of subunit vaccine formulations containing recombinant Hendra virus soluble G glycoprotein*

Trial no., horse identification	Hendra virus soluble G glycoprotein specification				Challenge, days after vaccination		Viral infectivity control	
	Source	Irradiation	Dose, μg				Species	No.
1							Horse	1
V1	293F HEK	No	100		21			
V2	293F HEK	No	100		21			
2							Guinea pig	4
V3	293F HEK	No	50		21			
V4	293F HEK	No	50		21			
V5	293F HEK	No	50		21			
3							Ferret	2
V6	CHO	Yes	100		21			
V7	CHO	Yes	100		21			
4							Ferret	2
V8	CHO	Yes	100		194			
V9	CHO	Yes	100		194			
V10	CHO	Yes	100		194			

*HEK, human embryonic kidney cells; CHO, Chinese hamster ovary cells.

### Immunization

All immunizations comprised two 1-mL doses administered intramuscularly 3 weeks apart, unless stated otherwise. In the efficacy studies, 7 horses (V1, V2, and V6–V10) received vaccine containing 100 μg of HeVsG glycoprotein/dose and 3 horses (V3–V5) received 50 μg of HeVsG glycoprotein/dose (Table 1).

### Animal Infection

Horses in the efficacy studies were exposed oronasally to 2 × 10^6^ 50% tissue culture infectious doses of a low-passage HeV isolate (Hendra virus/Australia/Horse/2008/Redlands). Horses V1–V7 were challenged 28 days after the second vaccination, and horses V8–V10 were challenged 194 days after the second vaccination. Horses V8–V10 were selected from 29 vaccinated horses in a larger field efficacy and safety study on the basis of temperament and for having the lowest serum neutralization titers in the group at the time. Overall, 4 efficacy tests were completed; 2 vaccinated horses were used in the first test, 3 were used in the second, 2 were used in the third, and 3 were used in the fourth. For the 4 tests, a pathogenicity control for the inoculum was provided by 1 horse (test 1), 4 guinea pigs (test 2), 2 ferrets (test 3), and 2 ferrets (test 4). Guinea pigs and ferrets each received 50,000 50% tissue culture infectious doses of the same virus preparation that was used in the horses; guinea pigs received the dose by intraperitoneal injection, and ferrets received the dose by the oronasal route. Experience has shown that these doses and routes of administration were expected to be lethal in >25% of guinea pigs and 100% of ferrets. Exposure conditions for 3 additional unvaccinated control horses were equivalent to those used in both vaccinated horses and the inoculum-control horse and have been described (*6*).

### Sample Collection and Analysis

During efficacy studies, nasal, oral, and rectal swab samples; urine and feces samples; and blood samples (in EDTA) were collected from the horses before virus exposure and then daily until the animals were euthanized. Swab samples were collected in duplicate into 1 mL of phosphate-buffered saline for virus isolation or into 800 μL of MagMax Lysis/Binding Solution (Ambion, Austin, TX, USA) for RNA extraction. For urine and EDTA blood samples, 100 μL of fluid was added to 260 μL of the lysis/binding solution. At postmortem examination, the following tissues were collected for viral genome detection, virus isolation, histopathology, and immunohistochemistry according to (*15*): adrenal gland, bladder, brain (including olfactory pole), cerebrospinal fluid, guttural pouch, heart, kidney, large intestine, liver, lung, lymph nodes (bronchial, inguinal, intermandibular, mandibular, renal), meninges, nasal turbinates, ovaries, pharynx, small intestine, spinal cord, spleen, sympathetic nerve, trigeminal ganglion, and uterus. The following analyses were conducted as described (*15*): quantitative reverse transcription PCR for the detection of the HeV N gene, histology, immunohistology, serum neutralization test, and virus isolation.

## Results

**Figure 1:**
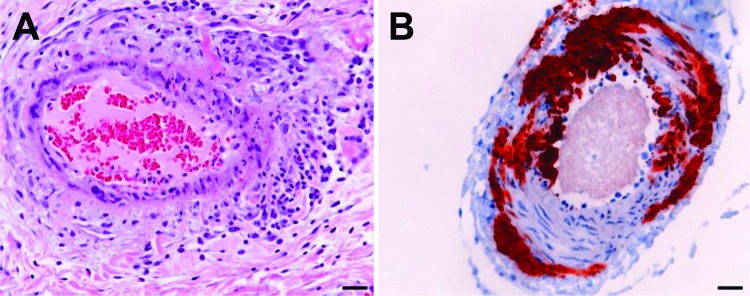
Histologic and immunohistologic findings in Hendra virus–infected horse tissue. A) Hematoxylin and eosin staining shows systemic vasculitis affecting the lung. B) Immunohistologic examination, using polyclonal rabbit anti-Nipah N protein, indicates Hendra virus antigen in a blood vessel in the brain. Scale bars represent 50 μm.

Vaccine efficacy in immunized horses was assessed against the clinical, virologic, and pathologic features of HeV infection in 4 unvaccinated control horses. Infection characteristics for 3 of these unvaccinated animals have been described (*6*); data from the fourth control animal was gathered as part of the current work. In that fourth control, onset of fever accompanied by a rising heart rate was noted on postchallenge day 6. On postchallenge day 7, the horse became clinically depressed, its temperature and heart rate continued to rise, and it was euthanized. Gross postmortem findings included pleural thickening and moderate dilation of the lymphatic vessels on the ventral 10 cm of the cardiac lung lobes. Histologic examination revealed systemic vasculitis affecting the lung (Figure 1, panel A), spleen, kidney, nasal epithelium, lymph nodes, and brain; alveolitis; and lymphadenitis. HeV antigen was identified in endothelial cells and vascular walls within lung, brain (Figure 1, panel B), nasal epithelium, lymph nodes, spleen, kidney, liver, myocardium, salivary gland, pharynx, small intestine, uterus, ovary, and adrenal gland, as well as in myocardial fibers and glomeruli.

Viral RNA from this fourth control horse was detected in nasal swabs collected on postchallenge day 3 (Table 2; summarized in Table 3) and also in blood collected immediately before the onset of fever. After onset of fever, but before development of other clinical signs of illness, HeV RNA was also detected in the oral swab sample. On the day of euthanasia, genome was detected in oral and nasal swab samples, blood, rectal swab, and urine samples; however, virus was not reisolated from any sample collected before postmortem examination. Viral RNA was detected in all tissues sampled at postmortem examination except cerebrospinal fluid. Reisolation of virus was attempted for all tissues: HeV was recovered from lung, submandibular lymph node, small intestine, large intestine, and adrenal gland.

**Table 2:** Quantitative reverse transcription PCR detection of Hendra virus N gene in samples collected daily from control horses*

Horse no., sample	log_10_ relative copy number of Hendra virus N RNA, dpc									
	0	1	2	3	4	5	6	7	8	9
1†										
Blood	−	−	−	−	−	−0.2	1.4	1.7		
Urine	−	−	−	−	−	−	−0.8	1.1		
Feces	−	−	−	−	−	−	−	−0.7		
Nasal swab	−	−	−0.3	−0.2	1.4	1.6	1.4	1.7		
Oral swab	−	−	−	−	−	−0.1	1.3	1.3		
2‡										
Blood	−	−	−	−	0.5	2.6	3.0			
Urine	−	−	−	−	−	0.3	1.7			
Feces	−	−	−	−	−	−0.05	2.0			
Nasal swab	−	−	1.2	2.0	1.6	3.5	2.5			
Oral swab	−	−	−	−	−	0.4	1.5			
3‡										
Blood	−	−	−	−	−	−	1.5	2.8	2.9	3.4
Urine	−	−	−	−	−	−	−	1.8	1.7	3.0
Feces	−	−	−	−	−	−	−	1.5	1.7	2.1
Nasal swab	−	−	1.7	2.5	1.2	2.4	3.0	3.8	3.7	2.0
Oral swab	−	−	−	−	−	−	−	1.9	1.9	2.3
4‡										
Blood	−	−	−	−	−	0.1	1.9	2.5	3.0	
Urine	−	−	−	−	−	−	0.07	0.5	2.1	
Feces	−	−	−	−	−	−	1.3	2.4	2.1	
Nasal swab	−	−	0.3	−	−	−	−	1.6	2.5	
Oral swab	−	−	−	−	−	−	0.2	1.2	1.6	

*Duplicate samples were obtained and tested by reverse transcription PCR. Cycle threshold values were converted to relative copy numbers by using a standard curve of a sample with a known copy number. dpc, days after challenge. − indicates a negative result; blank space indicates no sample was tested. †N gene data for horse 1 was obtained from the current study. ‡N gene data for horses 2–4 are unpublished data from a previous study (*6*).

**Table 3:** Summary of sample analysis data from 10 horses in the 4 efficacy trials*

Trial no, horse ID	Prechallenge antibody titer	Euthanized, dpc		Genome detection, sample, no. dpc								Viral infectivity control	
				PM tissue	Oral swab	Rectal swab	Nasal swab	Urine	Feces	Blood		Specimen	No. died/no. total
1												Horse	1/1
V1	512, 1,024	8		−	−	−	−	−	−	−			
V2	512, 1,024	9		−	−	−	−	−	−	−			
2												Guinea pig	1/4
V3	2,048, 4,096	7		−	−	−	−	−	−	−			
V4	128, 256	8		−	−	−	−	−	−	−			
V5	>4,096, >4,096	9		−	−	−	−	−	−	−			
3												Ferret	2/2
V6	>4,096, >4,096	7		−	−	−	−	−	−	−			
V7	>4,096, >4,096	8		−	−	−	−	−	−	−			
4												Ferret	2/2
V8	32, 32	7		−	−	−	−	−	−	−			
V9	16, 32	8		−	−	−	2–4, 7	−	−	−			
V10	16, 32	9		−	−	−	−	−	−	−			
*A 100-μg dose of Hendra virus soluble G glycoprotein was used in trials 1, 3, and 4; a 50-μg dose was used in trial 2. Genome was detected by PCR. ID, identification; dpc, days after challenge; PM, postmortem. − indicates a negative result.													

In a series of vaccine efficacy studies, 10 horses were immunized with HeVsG glycoprotein and then exposed to an otherwise lethal dose of HeV by the oronasal route. Each study also included a pathogenicity control for the virus inoculum. In the first of these, the pathogenicity control was the fourth control horse described above. Together with historical data gathered from 3 horses following their exposure to HeV under equivalent experimental conditions (*5*), data from this horse completed the requirements of the Australian Pesticides and Veterinary Medicines Authority for defining the horse infection model. In subsequent studies, guinea pigs or ferrets were used as pathogenicity controls to maximize the number of vaccinated horses that could be accommodated in the BSL-4 facility. These animals duly displayed signs, lesions, tissue antigen and viral genome distribution, and virus reisolation data consistent with acute HeV infection.

**Figure 2:**
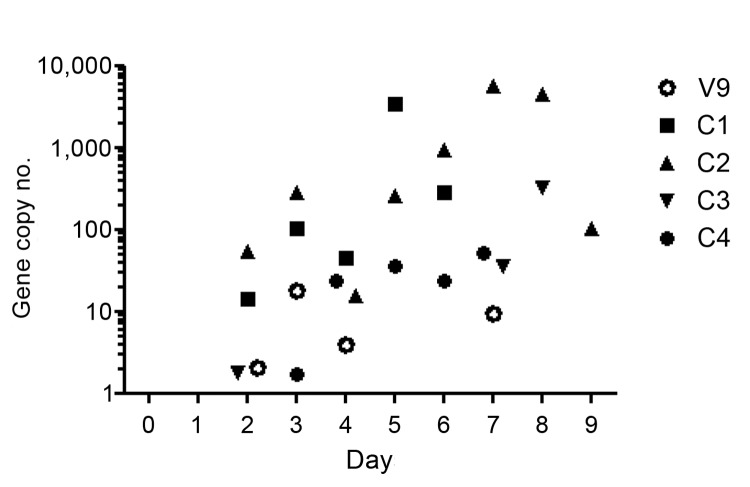
Scatter plot showing quantitation of the Hendra virus N gene in nasal swab samples from 1 vaccinated horse (V9) and 4 control horses (C1–C4); controls were challenged but not vaccinated. Days represent days after challenge.

In contrast to unvaccinated control horses, vaccinated horses remained clinically healthy during the observation period after exposure to HeV. Following elective euthanasia at the time of predicted peak viral replication, there was no gross or histologic evidence of HeV infection in vaccinated horses; all tissues examined were negative for viral antigen by immunohistochemistry; and viral genome was not recovered from any tissue, including nasal turbinates, pharynx, and guttural pouch (Table 3). For 9 of 10 vaccinated horses, viral RNA was not detected in daily nasal, oral, or rectal swab specimens or from blood, urine, or feces samples collected before euthanasia, and virus was not reisolated from any of these clinical samples. For 1 (V9) of 3 horses exposed to HeV 6 months after completing the vaccination course, low viral gene copy numbers were detected in nasal swab samples collected on postchallenge days 2–4 and 7 (Figure 2); this finding was consistent with self-limiting local replication. Virus was not reisolated from these samples.

Serum neutralization titers before HeV challenge ranged from 128/256 to >4,096 for horses V1–V7 when challenged 21 days after the second vaccination and from 16 to 32 for horses V8–V10 when challenged 6 months after the second vaccination (Table 3). At the time of euthanasia, no rise in antibody titer was detected in any vaccinated horse following exposure to HeV.

## Discussion

The formal launch of the HeV horse vaccine in November 2012 represents the culmination of multiple studies conducted in several animal infection models over the course of many years. Studies using Nipah virus in cats (*16*,*17*) and monkeys (*18*) and HeV in ferrets (*15*) provided strong evidence that a HeVsG glycoprotein subunit–based vaccine could prevent not only disease but often infection in animals exposed to otherwise lethal doses of Nipah virus or HeV. Where evidence of low-level virus replication did occur in secretions, it was transient and unaccompanied by the development of clinical illness, and virus was not isolated from the secretions. 

The henipavirus surface-expressed G glycoprotein has the critical role of initiating infection by binding to receptors on host cells, and antibodies directed against this protein can neutralize virus (*19*). Earlier reports have shown that passive immunotherapy with antibody to the G or F glycoprotein of HeV or Nipah virus alone can prevent fulminating disease (*20*): G glycoprotein–specific human monoclonal antibody prevented Nipah virus disease in ferrets (*21*) and HeV infection in African green monkeys (*22*); and F or G glycoprotein–specific monoclonal or polyclonal antibodies prevented HeV and Nipah virus disease in hamsters (*23*–*25*). Thus it is likely that, as seen for other paramyxoviruses with a viremic infection phase (e.g., measles and mumps), antibodies to the G and F glycoproteins play a major role in protection provided by HeVsG glycoprotein vaccination (*26*–*28*).

In the studies reported here, we show that 2 doses of a commercially formulated HeVsG glycoprotein subunit–based vaccine prevented infection in 7 of 7 horses exposed to HeV at least 21 days after the second vaccine dose; this finding is in contrast to that for unvaccinated control horses. Similar results were obtained for 2 of 3 horses exposed to HeV 6 months after vaccination. In the third horse, which also remained clinically healthy, evidence of HeV replication was limited to low-level transient detection of viral genome (but not virus) from the nasal cavity. In assessing the field significance of this observation, the following must be noted: the experimental horses were exposed to considerably higher levels of HeV than have been recovered from flying foxes (*1*), higher levels of viral genome were routinely found in the nasal secretions of nonimmunized horses, and all human infections have been acquired from animals in which clinical disease developed. It is reasonable to suggest that the higher transmission risk that is clearly associated with such horses is a consequence of not only increased viral load but also of the illness itself: it is the clinically ill horse that promotes increased human–animal contact through diagnostic investigations and administration of nursing care. We conclude that the level and pattern of virus replication in the 1 vaccinated horse do not meet the epidemiologic criteria presently associated with transmission of infection to humans.

In previous henipavirus vaccine efficacy studies in cats and ferrets, a neutralizing antibody titer of 32 was shown to be protective against the development of clinical disease (*17*). In the horse efficacy studies, the 3 horses with prechallenge antibody titers of 16 or 32 were similarly protected from clinical illness. However, we caution that any correlation between antibody titer at the time of exposure to virus and levels of subsequent protection against infection and disease is unlikely to be linear; it is possible that animals with even lower titers will have epidemiologically meaningful protection against HeV exposure occurring in the field, not least because of stimulation of immunological memory. Additional studies assessing the duration of protection are planned, and the outcome of these will further inform recommendations regarding booster vaccination.

As expected, initial uptake of the HeVsG glycoprotein subunit–based vaccine was strongest in the area with the highest perceived risk for HeV infection, namely coastal Queensland, Australia. In other regions where HeV infection of horses has not been reported, there is understandably more uncertainty regarding the value of vaccination as part of horse preventative health programs. Any reluctance to vaccinate horses against HeV that is based on assessment of risk is probably exacerbated by several factors, including the novelty of the vaccine roll-out process to the Australian horse industry, a (mistaken) perception that fast-tracking vaccine release involved overlooking key safety and efficacy issues, the lack of published data on safety in pregnant mares, reluctance of certain industry sectors to vaccinate because of import restrictions on HeV-seropositive horses, and cost. Although it is likely that each of these barriers will diminish over time, our experiences may assist the development of road maps to guide the future release of vaccines against BSL-4 pathogens that are associated with highly sporadic disease events and where the decision to vaccinate is in the hands of the persons whom vaccination was designed to protect.

Several recently emerged zoonotic viruses, including HeV, Nipah, Ebola, and Marburg viruses, are classified as BSL-4 agents because of their ability to cause severe illness or death in humans and because there have been no effective vaccines or postexposure treatments to protect against the diseases they cause. The vaccine against HeV (Equivac HeV) is a commercially deployed vaccine developed against a BSL-4 agent and is the only licensed treatment for henipavirus infection. 

Development of vaccines against BSL-4 agents for use in humans requires that the US Food and Drug Administration implement the animal rule, which requires that such vaccines first be tested for efficacy in at least 2 animal models (*29*). As a veterinary vaccine, Equivac HeV did not need to meet this requirement, and it was both cheaper and faster to produce than a vaccine intended for human use. At the same time, the vaccine is expected to provide a substantial health benefit to humans. In so doing, this vaccine encapsulates the spirit of a One Health approach, not just in terms of the interconnectedness of human and animal health but also with respect to environmental health. One consequence of the recent HeV outbreaks was a move to eradicate bat populations, despite their crucial environmental roles in pollination and reduction of the insect population. Successful deployment of the HeV vaccine, with a targeted reduction in the risk for acute disease events in horses and humans, should help reduce the current momentum toward the setting of control policies with potential adverse effects on the environment. Furthermore, the increasing evidence for henipaviruses and henipa-like viruses in bats in other areas (*30*–*32*) raises the possibility of future henipavirus outbreaks. The current HeVsG glycoprotein vaccine technology provides a platform for the rapid development of related vaccines to counter future emergent threats.
